# Rare Causes of Gastrointestinal Hemorrhage: A Case Series of Adult Duodenal and Jejunal Gastric Heterotopia

**DOI:** 10.7759/cureus.64604

**Published:** 2024-07-15

**Authors:** Venkata Vinod Kumar Matli, John Kirkikis, Gregory Wellman, Dustin Hadley, Ross M Dies, David F Dies

**Affiliations:** 1 Internal Medicine, Christus Highland Medical Center, Shreveport, USA; 2 Gastroenterology and Hepatology, Christus Highland Medical Center, Shreveport, USA; 3 Gastrointestinal and Liver Pathology, The Delta Pathology Group, Shreveport, USA; 4 Internal Medicine, Louisiana State University (LSU) Health New Orleans, New Orleans, USA

**Keywords:** polypoid mass, upper gi bleeds, obscure gastrointestinal bleeding, gastric heterotopia, gastrointestinal hemorrhage / etiology

## Abstract

Gastric heterotopia (GH) is a rare cause of gastrointestinal bleeding. GH of the small bowel is rare, and the duodenum is more commonly involved than the jejunum. Here, we present five cases of GH involving the duodenum and jejunum, with presentations including gastrointestinal bleeding, symptomatic anemia, and no symptoms.

A 63-year-old man presented with melenic stools but could not identify an obvious bleeding source during endoscopy. He was ultimately diagnosed with jejunal GH. A 70-year-old woman with melena and severe anemia had a duodenal bulb mass detected during endoscopy, which was histopathologically diagnosed as GH.

A 54-year-old woman experienced nausea, vomiting, and dysphagia. Endoscopy revealed esophagitis and a duodenal GH without malignancy. A 69-year-old woman incidentally had duodenal GH during evaluation for a lung mass, which was later diagnosed as an aggressive neuroendocrine tumor.

The fifth patient was an 83-year-old woman who was admitted for profound significant anemia. Upper endoscopy showed a round, 0.3 cm ulcer in the duodenum and a duodenal polyp with a tiny ulcer, and her histopathology was consistent with GH.

The exact mechanism of the action of GH remains unknown. Its clinical presentation is variable, gastrointestinal bleeding is rare, and diagnosis is based on histopathology only. Our case series emphasizes the need to include GH in the differential diagnosis of patients presenting with gastrointestinal bleeding, with or without other associated symptoms.

## Introduction

Heterotopia refers to organ displacement to a non-physiological site. Heterotopia can involve any gastrointestinal tract tissue. The most common lesions in the gastrointestinal tract are gastric heterotopia (GH), pancreatic heterotopia, and heterotopia of the Brunner’s glands. Heterotopia of the gastric mucosal tissue in the small bowel is a rare congenital disorder usually diagnosed during childhood [1]. The most common sites of GH are the esophagus and remnants of the omphalomesenteric duct (Meckel’s diverticulum) [1,2]. We present a case series comprising five adult individuals, with four of them diagnosed with duodenal GH (DGH) and one with jejunal GH (JGH).

## Case presentation

Case 1

A 63-year-old man, who had been experiencing melenic stools for the past week, was the first patient. The patient had a medical history of paroxysmal atrial fibrillation, cerebrovascular accident, essential hypertension, type-2 diabetes (T2DM), and coronary artery disease. The patient was on apixaban for the atrial fibrillation and stroke prophylaxis. One month before the presentation, the patient underwent elective left heart catheterization requiring drug-eluting stent placement in the left anterior descending artery and was advanced to dual antiplatelet therapy.

Anemia was noted during the patient's presentation, resulting in the initiation of treatment through intravenous iron infusions and two units of packed red blood cell (PRBC) transfusions. Following discharge, the patient underwent thorough endoscopic evaluation, including upper endoscopy, wireless capsule endoscopy, and colonoscopy. Surprisingly, none of these studies revealed evidence of the bleeding source.

The patient denied abdominal pain, fever, chills, loss of weight or appetite, nausea, vomiting, diarrhea, or bright red blood per rectum. Upon examination, his vital signs were stable. The abdomen was soft, non-distended, and non-tender, with normal bowel sounds. His stool guaiac test result was positive. His laboratory results were significant for a hemoglobin of 6.5 g/dl (reference range: 13.7-17.5 g/dl), and hematocrit of 28.5% (reference range: 40.1%-51.0%). The comprehensive metabolic panel (CMP) was normal. Computed tomography (CT) of the abdomen and pelvis without contrast was unremarkable, except for gallbladder stones with distension. He was transfused with two units of PRBC, and his hemoglobin level improved and remained between 8.5 and 9.0 g/dl until he was discharged.

**Figure 1:**
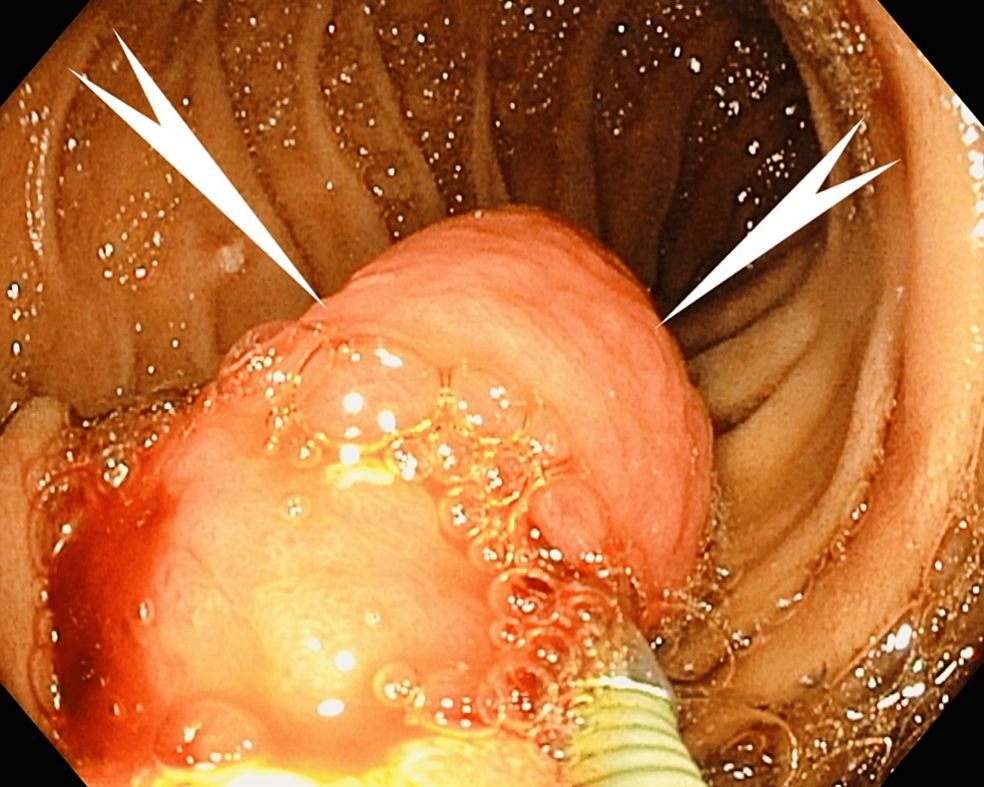
Case 1: Enteroscopy showing a 2.5-cm polypoid mass lesion in the region of the proximal jejunum (white pointed arrow)

**Figure 2:**
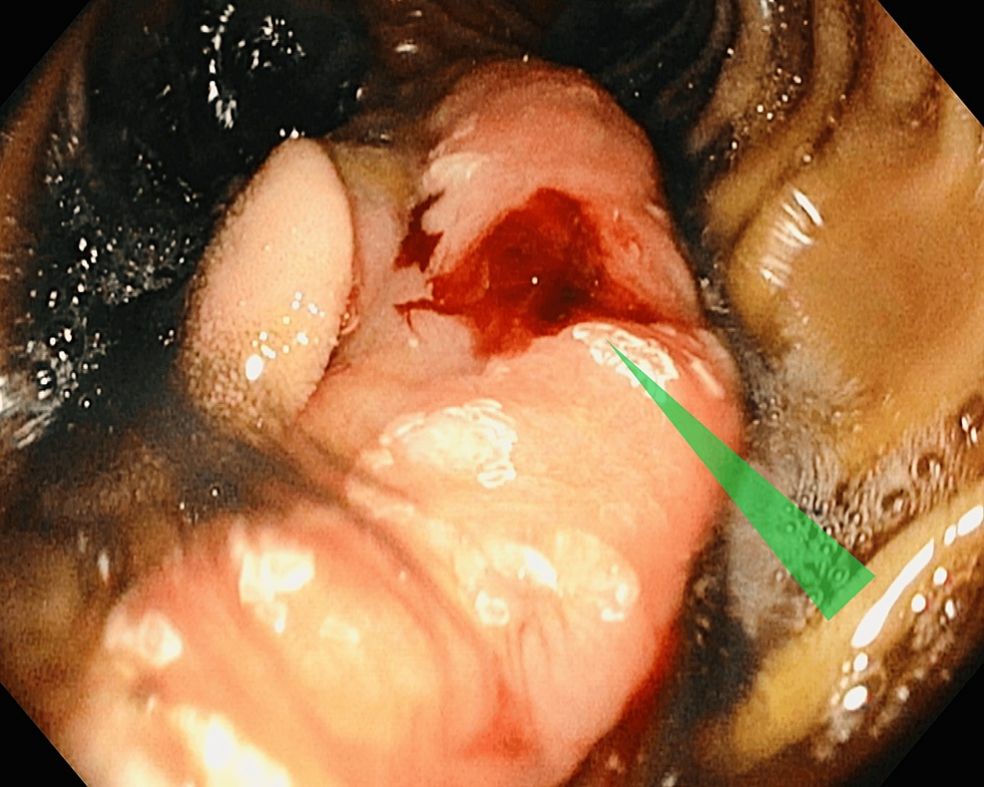
Case 1: Enteroscopy showing a polypoid mass in the proximal jejunum with mucosal oozing (green triangle)

**Figure 3:**
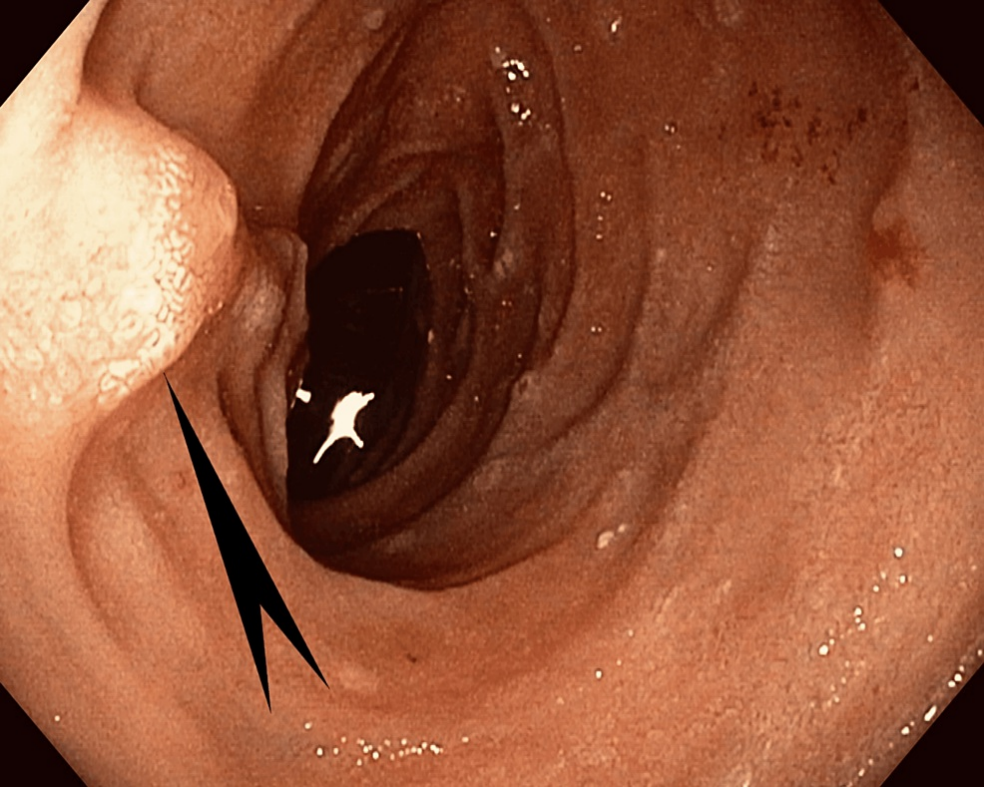
Case 1: Enteroscopy showing a second polypoid mass measuring 0.5 cm in the distal jejunum, 15 cm from the first mass (black pointed arrow)

As the findings of the patient’s outpatient endoscopy were unremarkable, he underwent a diagnostic enteroscopy. This study showed a 2.5-cm polypoid mass lesion with mucosal oozing in the proximal jejunum (Figures 1-3).

**Figure 4:**
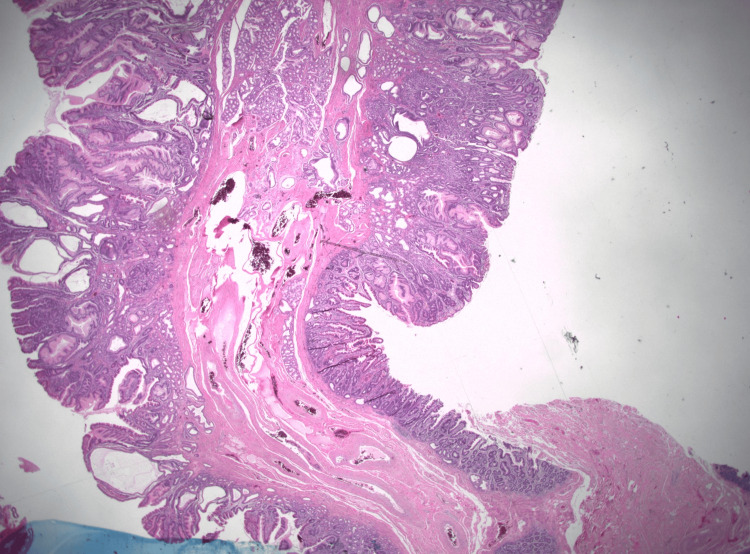
Case 1: Low-power photomicrograph showing a pedunculated duodenal polyp with features of mucosal prolapse (hematoxylin and eosin stain, 10.25X original magnification)

**Figure 5:**
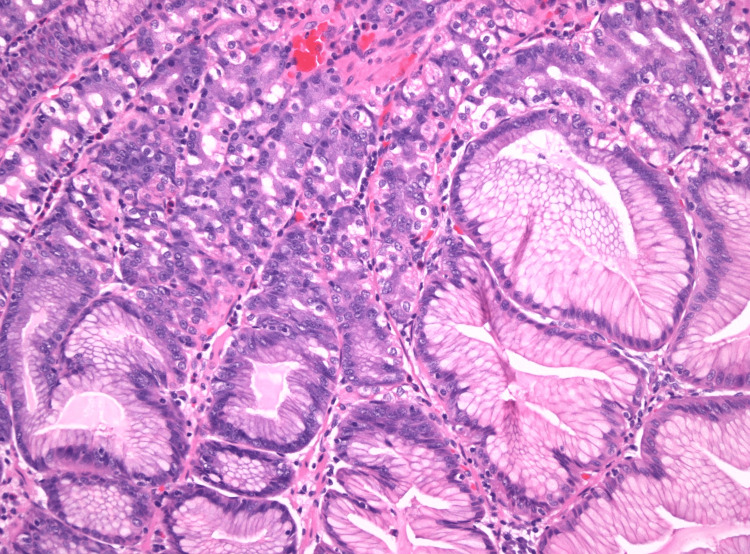
Case 1: Medium-power photomicrograph demonstrating heterotopic gastric oxyntic-type mucosa within the duodenal polyp (hematoxylin and eosin stain, 200X original magnification)

Biopsies were obtained for histopathological studies, and tattoos were placed to mark the lesions. Histopathological examination revealed GH with prominent polypoid gastric foveolar metaplasia and reactive epithelial changes (Figures 4, 5).

No evidence of dysplasia or malignancy was found. The patient was advised to continue clopidogrel and avoid apixaban as the risk exceeded the benefit. He was also counseled on avoiding over-the-counter non-steroidal anti-inflammatory medications. Subsequently, the patient was readmitted for acute calculus cholecystitis and successfully underwent cholecystectomy with jejunal resection. The postoperative course was uneventful.

Case 2

**Figure 6:**
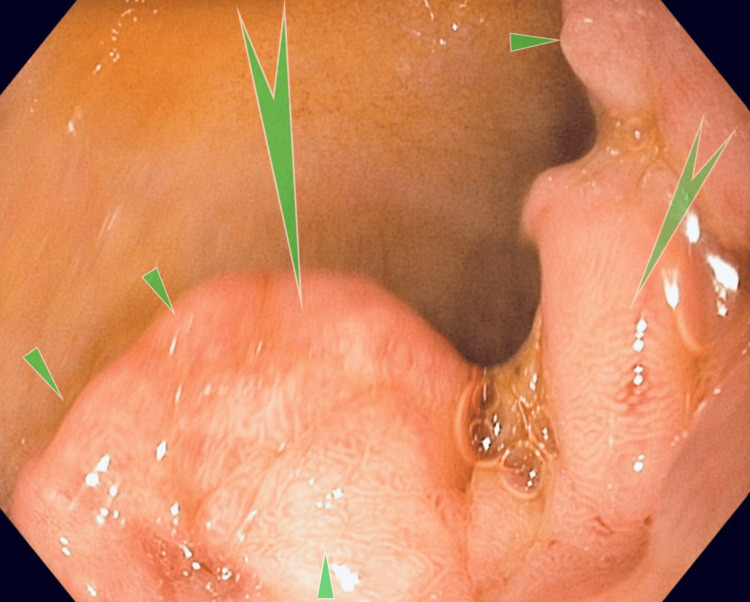
Case 2: Esophagogastroduodenoscopy showing a fleshy polypoid mass in the duodenal bulb (pointed green arrows and triangles). Fleshy tissue in the duodenal bulb covered at least half of the circumference extending from the superior posterior inferior aspect of the bulb.

The second patient was a 70-year-old woman whose medical history was significant for aortic valve replacement, coronary artery disease, and atrial fibrillation on apixaban therapy. She presented with progressive dyspnea and complaints of melenic stools for the prior four weeks. She was found to have low hemoglobin and was directed to the emergency room. Bidirectional endoscopy and wireless capsule endoscopy three months prior and a second esophagogastroduodenoscopy (EGD) one month prior did not reveal an obvious source of bleeding. Her vital signs were stable. She had a conjunctival pallor. Her abdominal exam was unremarkable. Stool guaiac test results were positive. Her laboratory test results were significant for hemoglobin (8.0 gm/dl) and hematocrit (24.3%), while her CMP was essentially normal. EGD showed fleshy tissue that covered at least half of the bulb circumference, extending from the superior aspect of the bulb (Figure 6).

**Figure 7:**
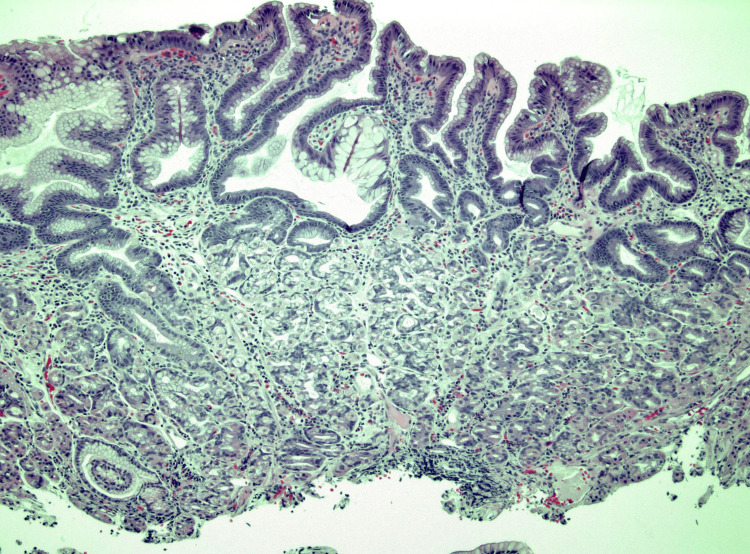
Case 2: Medium-power photomicrograph showing duodenal mucosa with surface foveolar metaplasia and heterotopic oxyntic-type gastric mucosa recapitulating negative gastric mucosa (100X original magnification, hematoxylin and eosin stain)

**Figure 8:**
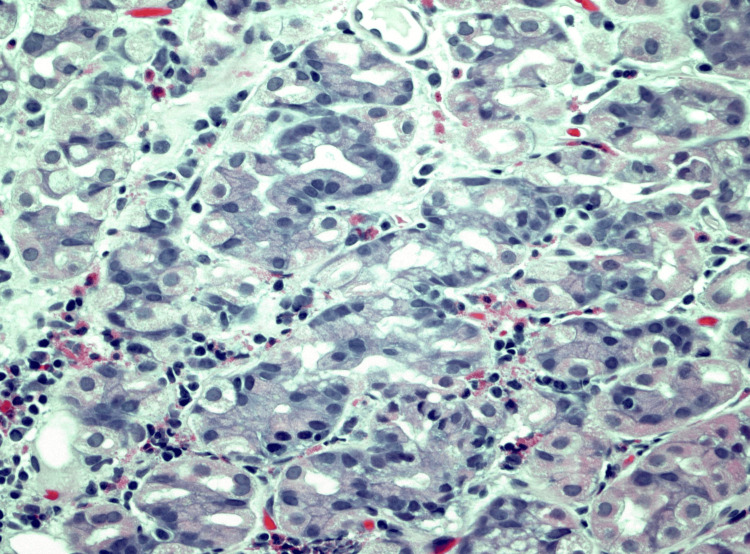
Case 2: High-power photomicrograph demonstrating the heterotopic gastric type oxyntic mucosa (400X original magnification; hematoxylin and eosin stain)

This image shows a discrete lesion marked by the arrow. No stigmata of bleeding were present. Histopathology of the tissue showed GH with foveolar hyperplasia (Figures 7, 8).

The patient was discharged home and advised to discontinue apixaban therapy as the risk exceeded the benefit; however, aspirin was continued. Unfortunately, the source of bleeding was not identified even after the third endoscopic study.

Case 3

**Figure 9:**
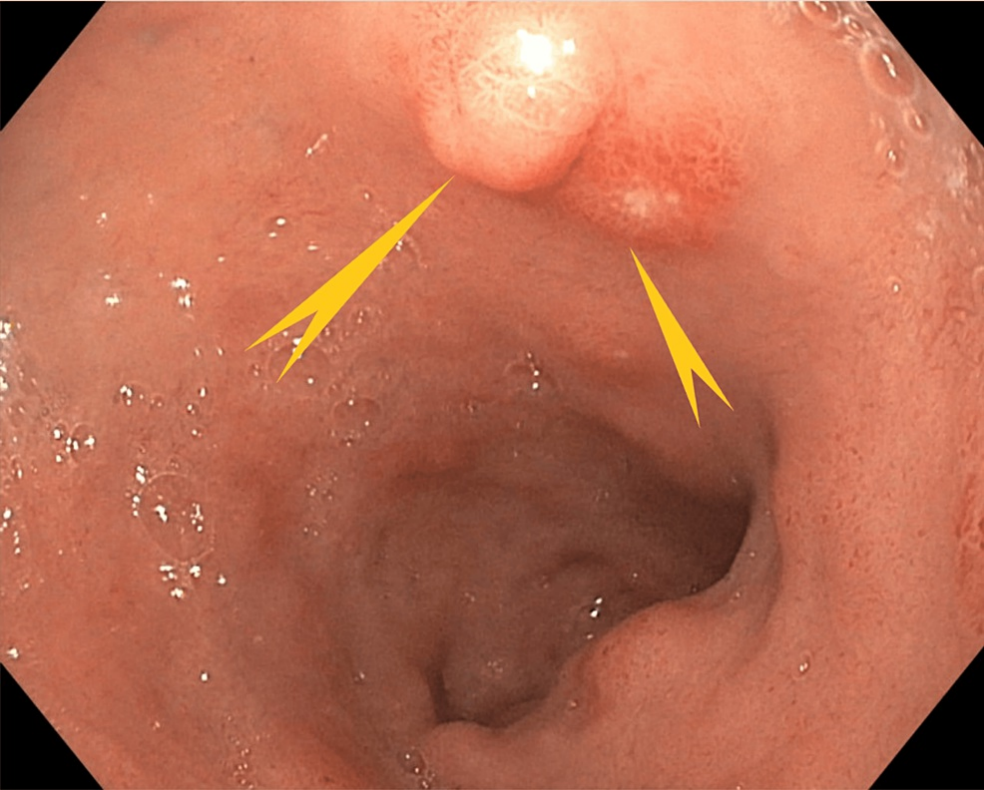
Case 3: Single medium semi-pedunculated polyp in the duodenal bulb (pointed arrow)

The third patient was a 54-year-old woman who presented with nausea, vomiting, and dysphagia. Upper endoscopy showed grade II esophagitis at the gastroesophageal junction and a single, semi-pedunculated polyp in the duodenal bulb (Figure 9).

**Figure 10:**
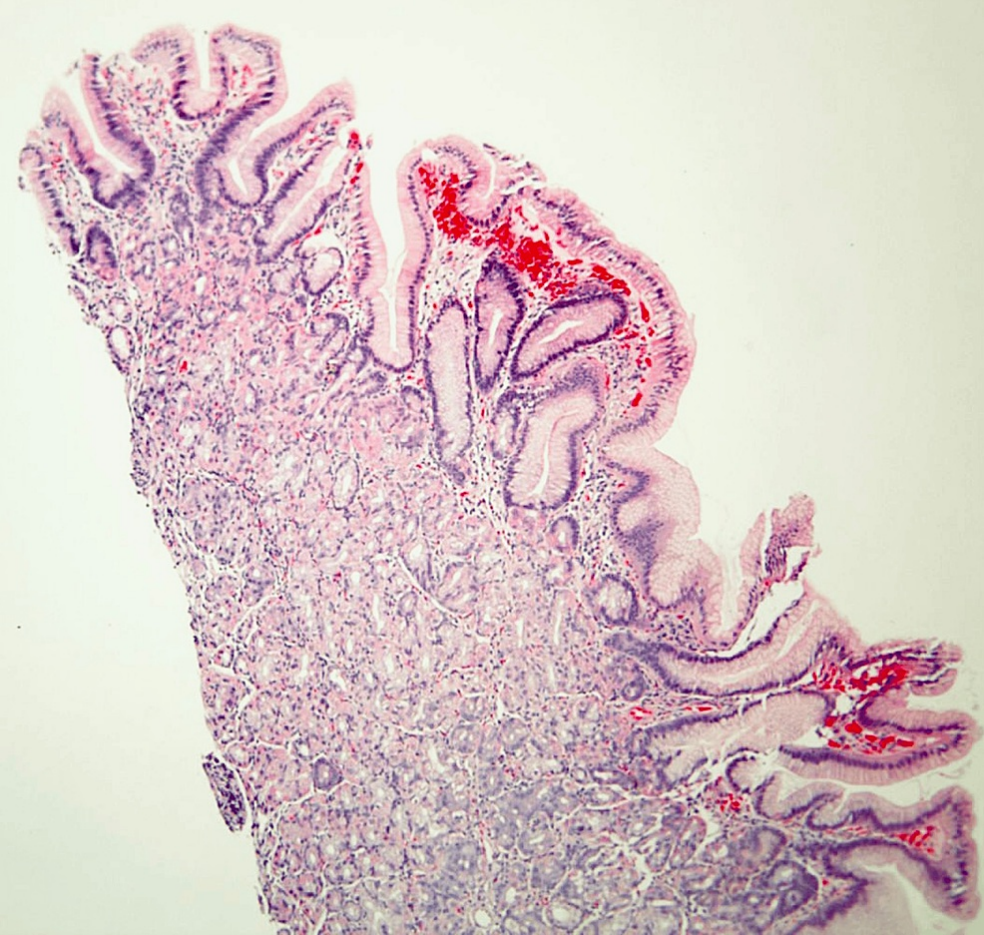
Case 3: Microscopic sections of this "duodenal polyp" show blunted villous architecture with the normal intestinal-type surface epithelium largely replaced by gastric foveolar-type epithelium, with only rare residual goblet cells still visible. The underlying small bowel crypts and Brunner glands are replaced by gastric fundic-type glands, with mild hemorrhage and nonspecific inflammation. The overall findings are consistent with gastric heterotopia.

Histopathology showed GH but was negative for dysplasia or malignancy (Figure 10). The patient’s symptoms improved with proton pump inhibitor therapy. This was an incidental finding of asymptomatic duodenal GH. The patient did not complete the scheduled follow-up.

Case 4

**Figure 11:**
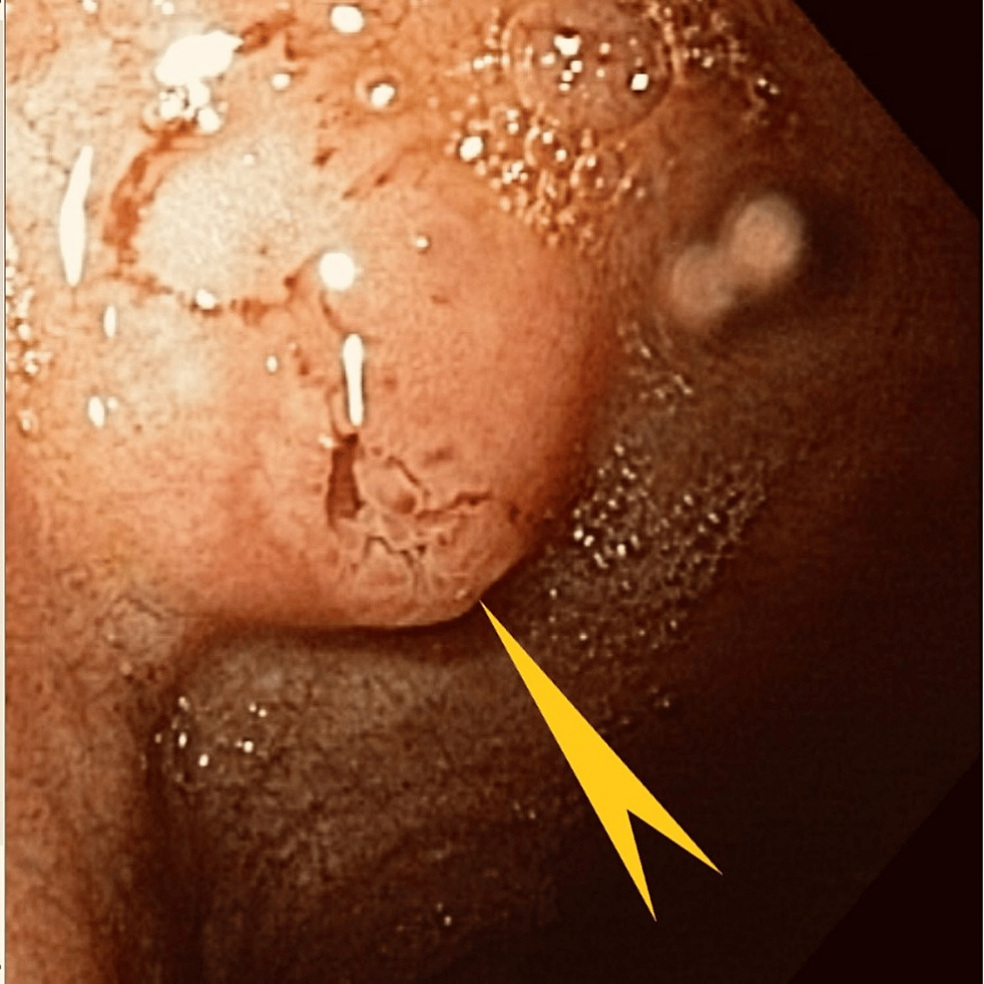
Case 4: Upper endoscopy showing nodular polyp in the duodenum (pointed yellow arrow)

The fourth patient was a 69-year-old woman whose medical history was significant for essential hypertension, T2DM, and chronic tobacco abuse. She was evaluated for a right lower lobe mass found on contrast-enhanced CT of the abdomen and pelvis. CT also showed hyperdense lesions in the liver, suggestive of metastatic disease. Unfortunately, histopathological studies of the liver lesions showed an aggressive small-cell neuroendocrine tumor of pulmonary origin. During hospitalization, the patient developed diffuse abdominal pain and required upper endoscopy. Her vital signs were stable, and the physical exam was unremarkable. Lab showed a normal complete blood count and a normal CMP except for mildly elevated aspartate aminotransferase levels of 119 u/L (reference: 15-37 u/L) and alkaline phosphatase level of 157 u/L (reference: 46-116 u/L). Upper endoscopy showed mild chronic gastritis and nodular polyps in the duodenum (Figure 11).

**Figure 12:**
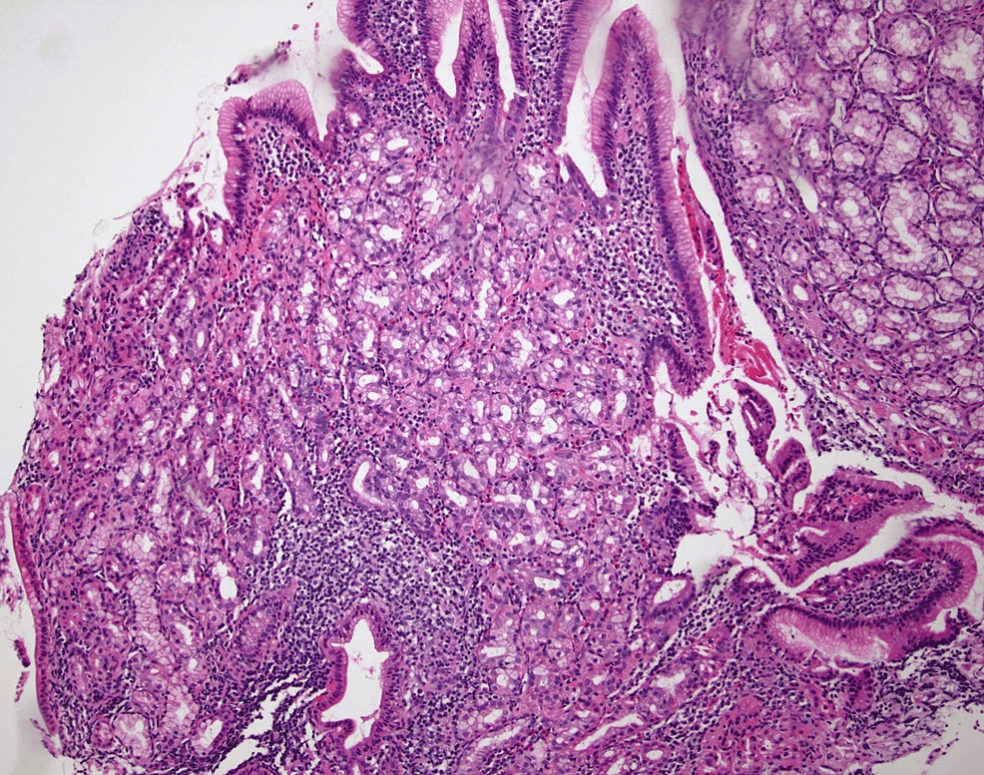
Case 4: Medium-power photo micrograph of duodenal mucosa demonstrating heterotopic gastric oxyntic-type glands adjacent to the duodenal mucosal epithelium (hematoxylin and eosin stain, 100X original magnification)

Histopathology showed benign GH mucosa in the biopsy of the duodenal nodule (Figure 12). Due to her poor prognosis, the patient was unfortunately discharged to hospice care.

Case 5

**Figure 13:**
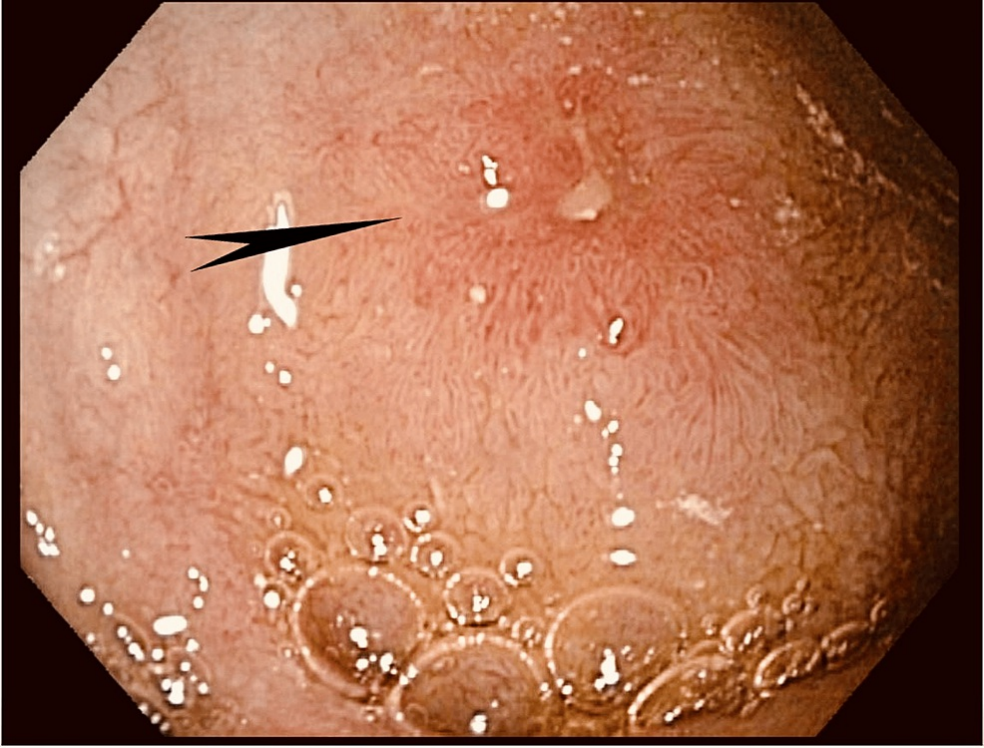
Case 5: Upper endoscopy showing duodenal polyp with a tiny ulcer over it (pointed black arrow)

**Figure 14:**
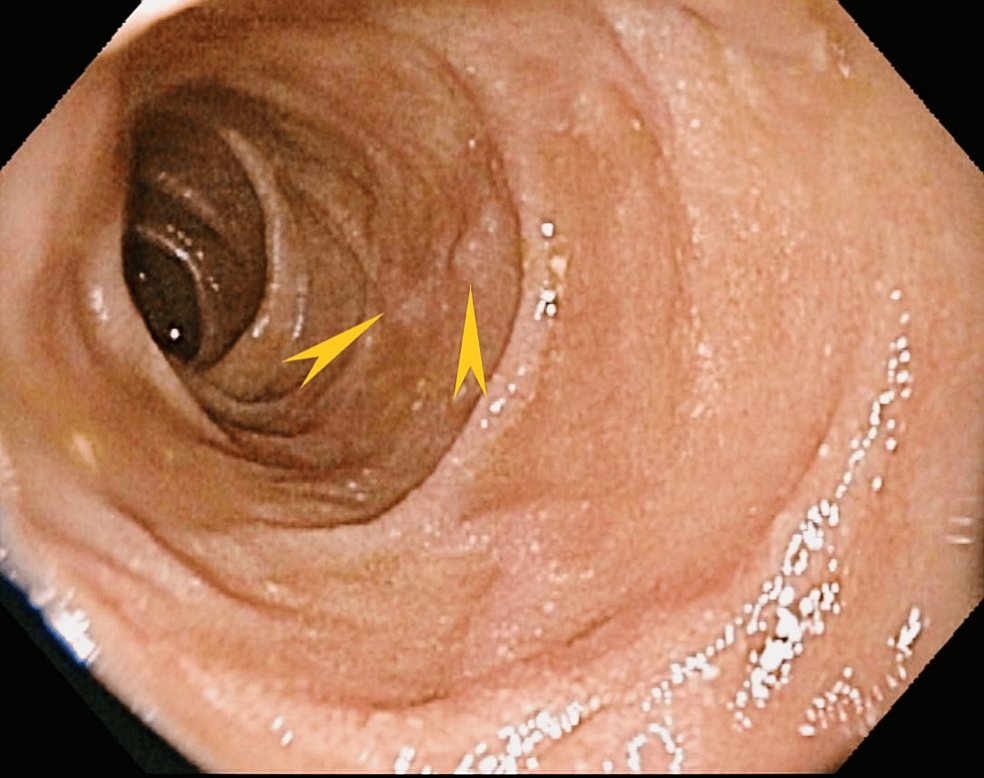
Case 5: Upper endoscopy showing duodenal polyp with a tiny ulcer over it (pointed yellow arrows)

The fifth patient was an 83-year-old woman with a medical history of T2DM, essential hypertension, and chronic kidney disease (CKD) stage 3. She was admitted for profound significant anemia with a hemoglobin level of 4.6 g/dL and a hematocrit level of 14.8%. The patient was transfused with two units of PRBC. Her vital signs were stable, and her physical exam only showed conjunctival pallor. Her CMP was within normal limits except for a low estimated glomerular filtration rate, which was proportional to CKD stage 3. Upper endoscopy showed a round, 0.3 cm ulcer in the duodenum and a duodenal polyp with a tiny ulcer over it (Figures 13, 14).

**Figure 15:**
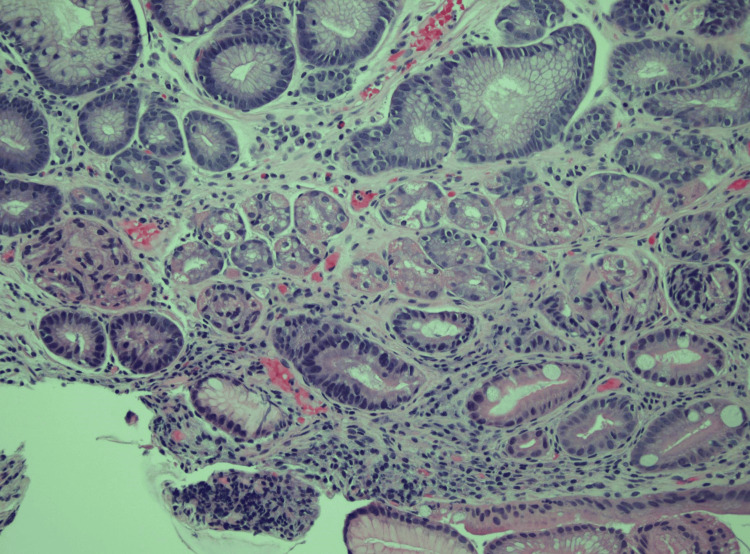
Case 5: Medium-power photo micrograph of duodenal mucosa demonstrating heterotopic gastric oxyntic-type glands adjacent to the duodenal mucosal epithelium (hematoxylin and eosin stain, 100X original magnification)

The histopathological examination was consistent with GH and was negative for *Helicobacter pylori*. The patient received treatment with proton pump inhibitors and iron supplements. At the follow-up appointment, the patient's hemoglobin improved to 8.5 g/dL. A surveillance endoscopy was scheduled one year from the follow-up appointment (Figure 15).

All these cases are summarized in Table 1.

**Table 1:** Summary of five cases

Case	Age (years)	Sex	Site of heterotopia	Clinical presentation	Management
1	63	M	Jejunum	Melena	En bloc jejunal resection
2	70	F	Duodenum	Melena	Partial Whipple procedure
3	54	F	Duodenum	Asymptomatic incidental finding	Observation
4	69	F	Duodenum	Abdominal pain	Hospice care due to coexistent metastatic pulmonary neuroendocrine tumor
5	83	F	Duodenum	Symptomatic anemia	Medical management

## Discussion

GH is an uncommon source of gastrointestinal bleeding. GH beyond the ligament of Treitz is exceptionally rare, except for Meckel’s diverticulum, which has an estimated prevalence of 2% in adults [1]. The first reported case of GH involving the ileum dates back to 1912 when Poindecker described a nine-year-old girl with ileus symptoms [3]. Subsequently, in 1932, Barták published the first case of jejunal GH (JGH) [4].

Proximal to the ligament of Treitz, GH is relatively more common. Taylor’s study [5] in 1927 introduced the concept of duodenal GH (DGH), which is often found as multiple small polyps within the duodenal bulb. Agha et al. [6] histologically confirmed 17 patients with duodenal bulb GH, with the duodenal bulb being the primary location, although the distal duodenum may also be involved [7]. The exact mechanism underlying GH remains unknown, but some literature suggests a link to the transforming capability of epithelial cells in the small bowel [1].

GH typically presents incidentally and asymptomatically, with diagnosis relying solely on histopathological studies. Endoscopic appearance does not provide diagnostic clues. JGH, although usually asymptomatic, can manifest with abdominal pain, nausea, vomiting, intestinal obstruction, ulceration, bleeding, perforation, and strictures. Our case series included patients with fleshy, polypoid mass lesions in the jejunum and duodenum as well as nodular, rounded polyps (one with semi-pedunculated polyps). Histopathology consistently revealed heterotopic oxyntic-type gastric mucosa. Lee et al. [8] reported tumorous mass lesions in JGH patients, including serpiginous patterns. Our patient with JGH also presented with a polypoid mass and bleeding on endoscopy. Metaplasia, distinct from heterotopia, occurs in response to harmful gastric acids or inflammation.

Heterotopia, a congenital phenomenon, is diagnosed histopathologically [9]. A retrospective histopathologic study by Terada, consisting of 158 cases, reported two types of heterotopic gastric mucosal tissue. The first type containing gastric glands, including chief and parietal cells, was found in 82% of esophageal and 73% of duodenal GH patients. The second type comprised foveolar epithelium in 18% of esophageal and 27% of duodenal GH patients; this shows that esophageal GH and duodenal GH are common, with gastric heterotopia found in approximately 9% of the biopsy specimens [10].

Recently, diagnostic and therapeutic flexible endoscopy, such as push enteroscopy, intraoperative enteroscopy, and double-balloon enteroscopy (DBE), has gained significant momentum for the diagnosis of obscure gastrointestinal bleeding. Traditional endoscopy is insufficient to study small intestinal pathology beyond the ligament of Treitz. DBE provides high-resolution images, thereby allowing therapeutic interventions [11,12]. While traditional endoscopy diagnoses most GH cases of the foregut, small bowel endoscopy is necessary to diagnose jejunal GH.

Our case series emphasizes the need to include GH in the differential diagnosis when patients present with gastrointestinal bleeding. This will ensure timely diagnosis and minimize complications and mortality. Management includes resection of the lesions through endoscopy or surgery. Surgical resection is usually preferred for DGH and JGH to reduce complications resulting from associated anatomic and histological characteristics, such as wall thickness and high vascularity. However, size of the lesions, their location, and the severity of symptoms will dictate the management. Our patient with JGH underwent laparotomy with en bloc jejunal resection.

Table 2 provides a review of several reported cases of JGH in adults. Of the eight studies, only two presented with gastrointestinal bleeding [13,14]. Four patients presented with abdominal pain [8,15-18], one of which was diagnosed with intussusception [17,19] and one with intestinal obstruction [20]. In this review, the majority of patients required surgical resection through laparotomy, and only one patient who presented with bleeding underwent endoscopic resection [14].

**Table 2:** List of studies reporting various heterotopia

Study	Age (years)		Sex	Presenting complaint/diagnosis	Endoscopic/intraoperative findings	Management	
Ali et al. [13]	23		M	Melena, nausea, vomiting	Jejunal diverticulum with mucosal plaques	Laparotomy and endoscopic wedge resection	
Nasir et al. [14]		31	M	Postprandial abdominal pain and hematochezia	Polypoid mass at the duodenojejunal junction	Endoscopic resection	
Khan et al. [16]	36		F	Abdominal pain and vomiting	Polypoid lesion	Laparoscopic resection	
Lee et al. [8]	25		M	Abdominal pain postprandial and vomiting	Elongated, serpiginous mucosal tumors	Surgical resection	
Nwanze et al. [17]	24		M	Abdominal pain and intussusception	Protruding polypoid mass of the jejunum	Emergent surgical resection	
Vani et al. [18]	24		M	Abdominal pain and peritonitis	Jejunal strictures and perforation	Emergent exploratory laparotomy and resection	
Murshed et al. [19]		33	M	Intussusception	Intraluminal polypoid lesion from the ileocecal valve		Ileal segment resection with side-to-side ileo-ileal anastomosis
Mandrekar et al. [20]		22	F	Intestinal obstruction	Polypoid mass		Emergent laparotomy and resection

## Conclusions

GH is an uncommon cause of gastrointestinal bleeding, and its precise mechanism remains elusive. The clinical presentation of GH varies, and gastrointestinal bleeding is infrequent. Diagnosis relies solely on histopathological studies. Our case series underscores the importance of considering GH in the differential diagnosis when patients present with abdominal pain, gastrointestinal bleeding, or incidentally discovered endoscopic polypoid findings. Early recognition facilitates timely diagnosis and minimizes complications and mortality. Management options range from clinical observation to surgical resection, depending on the size, location, and severity of the symptoms associated with the lesion.
